# Control over the transverse structure and long-distance fiber propagation of light at the single-photon level

**DOI:** 10.1038/s41598-019-45082-6

**Published:** 2019-06-21

**Authors:** D. Cruz-Delgado, J. C. Alvarado-Zacarias, H. Cruz-Ramirez, J. E. Antonio-Lopez, S. G. Leon-Saval, R. Amezcua-Correa, A. B. U’Ren

**Affiliations:** 1 Instituto de Ciencias Nucleares, Universidad Nacional Autónoma de México, Apartado Postal 70-543, 04510 Cd.Mx. Mexico City, Mexico; 2 CREOL, The College of Optics and Photonics, the University of Central Florida, Orlando, Florida 32816 USA; 3 Sydney Astrophotonic Instrumentation Laboratory, School of Physics, University of Sydney, NSW 2006 Sydney, Australia

**Keywords:** Quantum optics, Other photonics

## Abstract

Quantum entanglement is arguably the cornerstone which differentiates the quantum realm from its classical counterpart. While entanglement can reside in any photonic degree of freedom, polarization permits perhaps the most straightforward manipulation due to the widespread availability of standard optical elements such as waveplates and polarizers. As a step towards a fuller exploitation of entanglement in other degrees of freedom, in this work we demonstrate control over the transverse spatial structure of light at the single-photon level. In particular we integrate in our setup all the technologies required for: (i) fibre-based photon pair generation, (ii) deterministic and broadband single-photon spatial conversion relying on a passive optical device, and (iii) single-photon transmission, while retaining transverse structure, over 400 m of few-mode fibre. In our experiment, we employ a mode selective photonic lantern multiplexer with the help of which we can convert the transverse profile of a single photon from the fundamental mode into any of the supported higher-order modes. We also achieve conversion to an incoherent or coherent addition of two user-selected higher order modes by addressing different combinations of inputs in the photonic lantern multiplexer. The coherent nature of the addition, and extraction of usable orbital angular momentum at the single-photon level, is further demonstrated by far-field diffraction through a triangular aperture. Our work could enable studies of photonic entanglement in the transverse modes of a fibre and could constitute a key resource quantum for key distribution with an alphabet of scalable dimension.

## Introduction

The generation, application and study of custom light fields with structured intensity, polarization and phase, i.e. structured light, is emerging as a key resource in all areas of optical research ranging from imaging to telecommunications^1^. The exploitation of structured light in the context of quantum entanglement and photon pair generation systems is no doubt an exciting pathway for future quantum photonics applications.

Photon pairs generated by spontaneous parametric processes have been at the heart of many experiments related to fundamental tests of quantum mechanics^2^ and to the implementation of various quantum-enabled technologies^3–5^. While spontaneous parametric downconversion (SPDC) in *χ*^(2)^ materials^6^ is an established means for the generation of photon pairs, the spontaneous four wave mixing (SFWM) process^7^, particularly involving fibre implementations, has gained prominence as an alternative with several important advantages including compatibility with existing fibre optic networks, as well as a greater scope for quantum state engineering^8^.

Concentrating on photons as the physical system of interest, entanglement may reside in any of the polarization, frequency-time and transverse position-momentum degrees of freedom. Furthermore, recent research on quantum states has explored discretized frequency-time in the form of time-bin entanglement^9^, and discretized transverse position-momentum, in the form of orbital angular momentum entanglement^10,11^, and it has also been shown that quantum states may be hyper-entangled^12^, i.e. involving multiple degrees of freedom. Many entanglement experiments to date have relied on the polarization degree of freedom^13^, largely because of the relative ease with which polarization states may be prepared and controlled using readily available components including wave plates and polarizers. It is comparatively more difficult to control the frequency-time and transverse momentum-position degrees of freedom in structured light at the quantum level. In our previous work we were interested in non classical light sources which rely on the discretized spatial structure obtained in a few-mode fibre^14^. Such an approach is in principle scalable, as controlled by the number of supported modes in the fibre, in contrast to polarization entanglement in which the Hilbert space for each photon is limited to a dimensionality of 2. While a few mode fibre, when used as a SFWM photon-pair source, can generate states which exhibit entanglement in transverse modes (i.e. spatial domain)^14^ it is typically challenging to carry out conclusive entanglement tests, which would require the availability of wave plate and/or polarizer analogues for the transverse spatial degree of freedom^15,16^. This serves as an important motivation for the current work, in which we demonstrate the ability to convert the single-photon transverse structure from the fundamental mode of a single mode fibre to the coherent addition of two user-selected fibre higher order modes. In particular, we demonstrate the operation of a mode selective photonic lantern (MSPL) spatial multiplexer as a half wave plate analogue in the spatial degree of freedom with functionality at the single photon level. This approach could enable future progress in the study and exploitation of quantum entanglement in the spatial domain.

In this paper we show the integration of all of the technologies required for: (i) fibre-based generation of photon pairs, (ii) *deterministic* and *broadband* conversion of the transverse mode structure, relying on a passive optical device, of one of the photons in each pair, heralded by the detection of its sibling, and (iii) propagation of the heralded single photon over a considerably long stretch of fibre. We thus demonstrate in a single setup many of the ingredients required for implementations of quantum information processing protocols based on the transverse mode degree of freedom in few-mode fibres.

## Mode Selective Multiplexers

**Figure 1:**
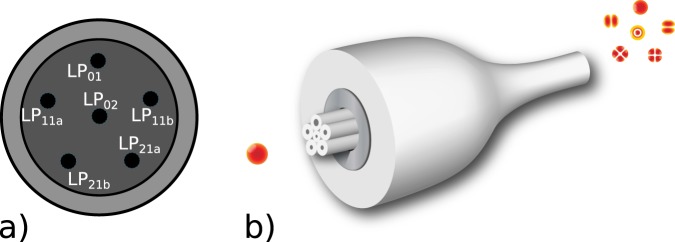
Schematic of 6 mode selective MSPL. (**a**) Transverse cross-section showing the 6 single-mode fibre array. (**b**) Adiabatic waveguide transition and spatial mode conversion.

We achieve transverse-mode control of the structured light at the single-photon level employing a photonic lantern, which is a passive fibre component that enables efficient conversion of multi-mode light into multiple single-mode signals^17–20^, and vice versa, originally developed for applications in the field of astrophysics^20,21^. Recently, they have been successfully used as efficient spatial-multiplexers in multi-mode optical communications research^18,22–24^. In a photonic lantern, multiple single-mode fibre (SMF) inputs are adiabatically tapered inside a low refractive index capillary to create a multi-mode waveguide at the taper waist. Within the taper transition, light propagating in single-mode waveguides adiabatically transitions into an orthogonal combination of the multi-mode waveguide modes at the taper end. Non-mode selective, or scrambling, devices can be fabricated using identical fibres^18,22^. In contrast, mode selectivity can be achieved exploiting dissimilar fibres for which the corresponding fundamental modes have distinct propagation constants so that each SMF excites exactly one spatial mode. Photonic lanterns are now considered as one of the most versatile mode multiplexers for next-generation multimode optical communication systems, by providing low insertion loss, low mode dependent loss and broad operational bandwidth^25^. Besides, MSPL’s can be scaled to a larger number of modes and can be easily spliced to a transmission fibre^22,26,27^. In our experiments we use a 6-mode photonic lantern (PL) designed for selectively generating the *LP*_01_, *LP*_11*a*,*b*_, *LP*_21*a*,*b*_ and *LP*_02_ modes in the 600 nm to 800 nm spectral range^28^, compatible with our photon-pair source. A schematic of the MSPL is shown in Fig. 1 in which we depict its structure consisting of six SMF inputs, with dissimilar core size, inside a low refractive index capillary.

## Results and Discussion

In our experiment, we employ a photon pair source based on the spontaneous four wave mixing (SFWM) process in a bowtie birefringent fibre. While this source, described previously in^14,29^ is capable of producing photon pairs in a coherent superposition of three distinct SFWM processes, in this work we spectrally post-select the process with the highest conversion efficiency, which involves all four waves which participate in the process propagating in the fundamental *LP*_01_ mode. In order to isolate a single photon from each pair, to be shaped using our MSPL technology in preparation for an arbitrary subsequent experiment (e.g. QKD or interaction with a cloud of cold atoms), we employ a *heralding* process in which direct detection of one of the photons in a given pair, say the idler, with an avalanche photodiode *heralds* its sibling photon. We subject the *heralded* single photon to transmission through the photonic lantern, as described below, and are thus able to deterministically convert its transverse wavevector intensity as verified with an ICCD camera, triggered by the detection of the *heralding* photon.

**Figure 2:**
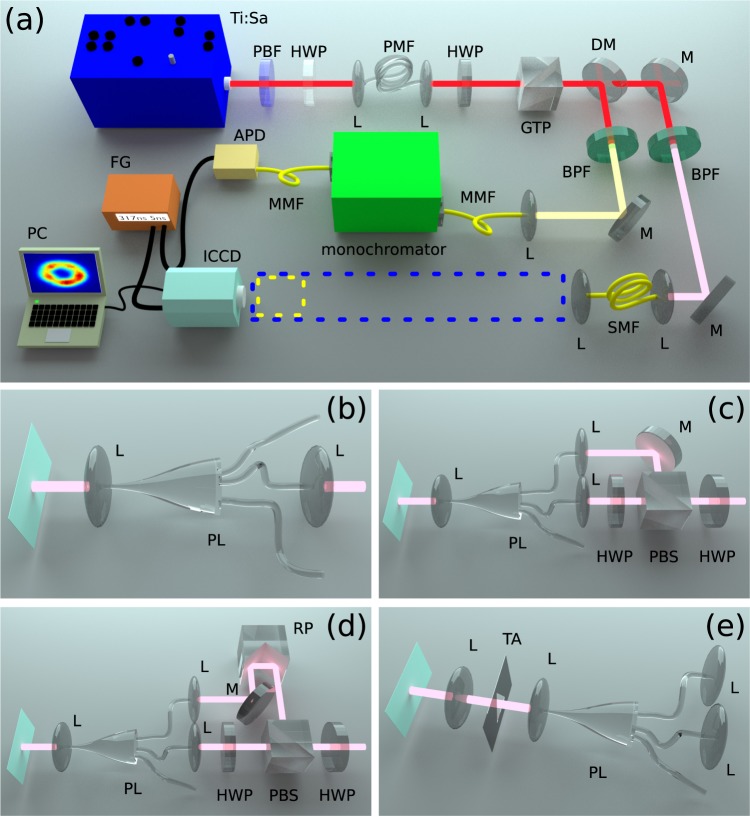
Schematic of experimental setup. (**a**) Photon-pair generation and detection. The signal-photon manipulation apparatus (dotted box in panel a), used in our experiments is shown the remaining panels. Ti:Sa: Titanium Sapphire pump laser, BPF: Band pass filter, HWP: Half wave plate, L: Lens, PMF: Polarization maintaining fiber, GTP: Glan-Thompson polarizer, DM: Dichroic mirror, M: Mirror, SMF: Singlemode fiber, MMF: Multimode fiber, monochromator: Czerny-Turner monochromator, APD: Avalanche photodiode, ICCD: Intensified CCD camera, FG: Function generator, PC: computer. (**b**) Single input excitation. (**c**) Incoherent dual-input excitation. d)Coherent dual-intput excitation. (**e**) Far-field diffraction technique.

Our experimental setup is shown in Fig. 2. A Ti:Sapphire laser, centered at 710 nm, with 76 MHz repetition rate and ~0.5 nm bandwidth was used. Spurious frequencies outside of this bandwidth are suppressed with a prism-based spectral bandpass filter (PBF). The pump, polarized parallel to the slow axis of the fibre, is coupled into a 12.5 cm length of birefringent bow-tie fibre (HB800C from Fibercore Ltd; PMF) using an aspheric lens of 8 mm focal length (L1); outcoupling is accomplished with an identical lens following the fibre (L2). In our SFWM source the generated photon pairs are orthogonally polarized with respect to the pump, so that the latter is suppressed using a Glan-Thompson polarizer (GTP).

Our SFWM source is spectrally non-degenerate, permitting the use of a dichroic mirror (DM) for splitting the photon pairs into two spatial modes. The idler photon, reflected by DM, is transmitted through a monochromator configured to transmit the window 636.6 ± 0.05 nm which corresponds to the desired idler mode and is detected with an avalanche photodiode. Each idler detection can herald the presence of a signal-mode single photon, transmitted by DM, centered at 802.2 nm. We have carried out a number of different experiments, each corresponding to a different configuration of the photonic lantern as indicated by the dotted box in Fig. 2(a). In our first experiment, the heralded single photon, with LP_01_ mode structure, is coupled into each of the six single-mode terminals of the six-mode MSPL in turn, as sketched in Fig. 2(b). This results in the deterministic conversion of the transverse mode of the heralded single photon from the fundamental mode *LP*_01_ to each of the six *LP*_01_, *LP*_11*a*,*b*_, *LP*_21*a*,*b*_ and *LP*_02_ modes, depending on the single-mode terminal used. This is verified by the detection of the heralded signal photon with an intensified charge coupled device (ICCD) camera operated in gated mode, with detection gates of 5.0 ns width defined by each idler photon detection.

**Figure 3:**
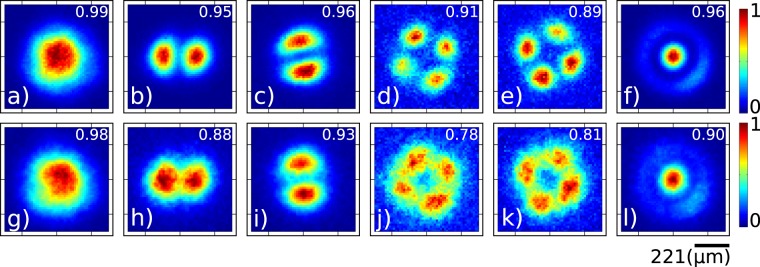
Measured transverse intensity at the single photon level for the heralded signal-mode photons when launched into each of the MSPL input terminals. Unconditioned data (above) and conditioned data (below).

We show our experimental results in Fig. 3, in which columns (a) through (f) correspond to each of the modes *LP*_01_, *LP*_11*a*,*b*_, *LP*_21*a*,*b*_ and *LP*_02_ obtained by coupling the signal photon into each of the six SMF input terminals. Note that while the first row corresponds to the unconditioned ICCD measurement, the second row represents the ICCD measurement of the heralded signal photon conditioned on the detection of a heralding idler photon. Note that conditioned counts are lower by a factor of around 50 with respect to unconditioned counts, reducing the observed data quality; longer acquisition times imply that our conditioned data measurements become more susceptible to any instabilities in our setup. Note also that we have calculated the correlation coefficient (covariance normalized by the product of standard deviations) between the theoretical and measured modes. The correlation values obtained are presented in the upper right corner of each panel; note that in all cases they are ≥0.9 (unconditioned) and ≥0.78 (conditioned).

A generalization to the experiment described above is to couple light into two (or more) different single-mode terminals of a MSPL device. In what follows we present a number of experimental measurements in which a heralded single photon is split into two spatial modes, each of which is coupled into a different single-mode terminal. A combination of half wave plate and polarizer is employed so as to control the splitting ratio into the two arms which are recombined at the MSPL forming essentially a Mach Zehnder interferometer, as sketched in Fig. 2(c). If the arm lengths differ by more than the coherence length of the input light, the two fields are summed incoherently; conversely, if the arm lengths are balanced to within the coherence length the two fields are summed coherently.

**Figure 4:**
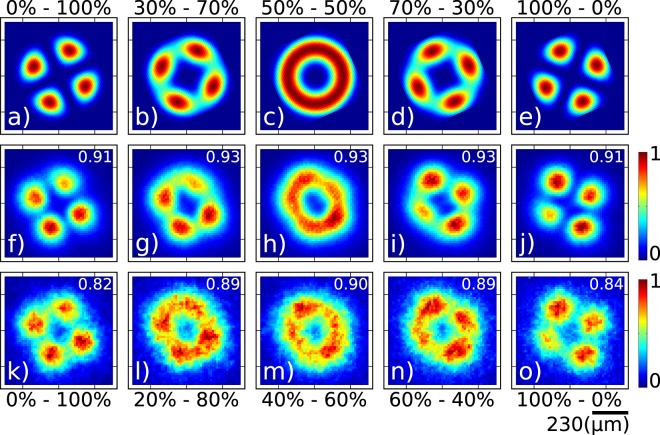
Measured transverse intensity at the single photon level obtained by launching the heralded single pohoton into two MSPL inputs, corresponding to modes *LP*_21*a*_ and *LP*_21*b*_, and involving an **incoherent** sum of these two modes. Numerical (first row), unconditioned data (second row) and conditioned data (third row). Splitting ratios shown atop each column.

In the incoherent case, if the modes corresponding to the two selected terminals are |*u*_1_〉 and |*u*_2_〉 with intensity fractions *α* = cos^2^*θ* and *β* = sin^2^*θ*, the output of the MSPL is given by the density operator $\hat{\rho }=\alpha |{u}_{1}\rangle \langle {u}_{1}|+\beta |{u}_{2}\rangle \langle {u}_{2}|$. While we can choose two arbitrary modes from the six available ones to be combined at the MSPL, it is particularly interesting to choose two with the same underlying topological charge (first subindex of the mode label). In Fig. 4 we show results for the incoherent sum of modes *LP*_21*a*_ and *LP*_21*b*_, as a function of the intensity splitting ratio in the two arms. The first row shows the calculated transverse intensity, i.e. *αI*_1_(*x*, *y*) + *βI*_2_(*x*, *y*), where *α* and *β* (with *α* + *β* = 1) represent the intensity fractions in the two arms and *I*_1_(*x*, *y*) and *I*_2_(*x*, *y*) represent the transverse intensity distributions for the two modes. Panels (a) through (e) represent the cases *α* = 0, *α* = 0.3, *α* = 0.5, *α* = 0.7, and *α* = 1.0 (with corresponding *β* values). The second row shows the measured transverse intensity at the single photon level unconditioned by the detection of the idler photon. The third row shows the measured transverse intensity at the single photon level conditioned by the detection of the idler photon. Note that by varying *α* from 0 to 1 we are able to continuously transition from mode *LP*_21*a*_ to *LP*_21*b*_, while obtaining a mode with an intensity null in the origin for *α* = 0.5. As for the single-terminal data in Fig. 3, we have presented the calculated correlation parameter values and presented them in the upper right-hand corner of each panel in the second and third rows. Note also that due to this imperfect correlation with the ideal modes, the experimental splitting ratios employed, chosen for visual agreement optimization with simulations, are not identical to the ones used in the simulations; they are as follows for the five columns: *α* = 0 ± 0.02, *α* = 0.2 ± 0.02, *α* = 0.4 ± 0.02, *α* = 0.6 ± 0.02, and *α* = 1 ± 0.02.

In the coherent case, for modes |*u*_1_〉 and |*u*_2_〉 corresponding to the two terminals, the outgoing mode is given by |*u*_1_〉 + *e*^*iϕ*^|*u*_2_〉. In this case, the intensity splitting ratios are left fixed at *α* = *β* = 0.5 and a delay line is introduced in one of the two arms which allows balancing the two arm lengths, as sketched in Fig. 2(d). Starting from balanced arms, small adjustments Δ*l* in the arm length difference result in variation of the phase *ϕ* = *k*Δ*l* (with *k* the wavenumber), which allows interferometric combination of the two modes. Two modes with the same topological charge in absolute value but differing in sign, are characterized by an identical transverse intensity as a function of position, yet with a different phase structure. Therefore, in the linear combination of two such modes, the amplitude can be factored, while it is the phases (or the functions depending on such phases) which combine in specific ways when performing the coherent addition of two modes. While it would of course be interesting to explore the coherent addition of two modes with differing absolute values of the topological charge as an extension of this work, our motivation here is obtaining a single-photon in an optical-vortex mode, i.e. with verifiable phase singularity. As this discussed below this can be accomplished by adding the *LP*_21*a*_ and *LP*_21*b*_ modes, or the *LP*_11*a*_ and *LP*_11*b*_ modes; in this paper we concentrate on the former pair of modes.

**Figure 5:**
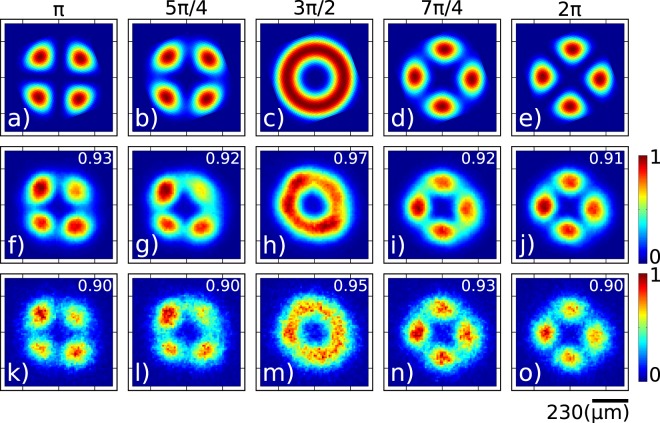
Measured transverse intensity at the single photon level obtained when launching the heralded single photon into two MSPL inputs, corresponding to modes *LP*_21*a*_ and *LP*_21*b*_, and involving a **coherent** sum of these two modes. Numerical (first row), unconditioned data (second row) and conditioned data (third row). Phase difference shown atop each column.

The first row of Fig. 5 shows the output mode obtained when *θ* is varied over a *π* radians range. The original modes *LP*_21*a*_ and *LP*_21*b*_ are obtained at the two extremes, while for *ϕ* = 270° (or *ϕ* = 90°) we obtain a mode with an intensity null in the center. The second row shows the measured transverse intensity at the single photon level unconditioned by the detection of the idler photon. The third row shows the measured transverse intensity at the single photon level conditioned by the detection of the idler photon. Note that while these results look very similar to those of the incoherent case, the experiment is completely different: in the first experiment we vary the relative intensities, while in the second experiment we leave the relative intensities fixed and vary the relative phase. As for the incoherent case, we have included correlation values between the ideal and measured modes in the second and third rows.

A single photon in mode *LP*_*lma*_, referred to as |*LP*_*lma*_〉, has a transverse field profile proportional to cos(*lϕ*) while a single photon in mode *LP*_*lmb*_ has a transverse profile proportional to sin(*lϕ*). While these modes are composed of superpositions of underlying vortices exp(*ilϕ*) and exp(−*ilϕ*) one way to access the implied orbital angular momentum (OAM) is to construct superpositions of |*LP*_*lma*_〉 and |*LP*_*lmb*_〉. In particular, let us define the following single-photon states with explicit topological charges *l* and −*l*

1 $$\begin{array}{rcl}|L{P}_{lm}^{\ast }\rangle  & = & \frac{1}{\sqrt{2}}(|L{P}_{lma}\rangle +i|L{P}_{lmb}\rangle )\\ |L{P}_{-lm}^{\ast }\rangle  & = & \frac{1}{\sqrt{2}}(|L{P}_{lma}\rangle -i|L{P}_{lmb}\rangle )\mathrm{.}\end{array}$$

While $|L{P}_{lm}^{\ast }\rangle$ has a field profile proportional to exp(*ilϕ*) leading to an explicit topological charge *l*, $|L{P}_{lm}^{\ast }\rangle$ has a field profile proportional to exp(−*ilϕ*) leading to an explicit topological charge −*l*. Note that we are able to prepare signal-mode single photons in these states by the coherent addition experiment reported above.

While in both the coherent mode addition and incoherent mode addition experiments we are able to obtain a ‘doughnut’-shaped transverse intensity pattern, see Figs 4(c) and 5(c), in order to experimentally distinguish between these two cases and to ascertain the presence of OAM in the coherent addition case, we need to employ an experimental strategy which responds to the underlying phase. One way to accomplish this is through the exploitation of far-field diffraction through a triangular aperture (TA)^30^. In the presence of OAM with topological charge *l* the far-field diffraction pattern shows a triangular pattern of intensity lobes, so that the lobe-count on one side of the resulting triangle is given by |*l*| + 1 for topological charge *l*.

**Figure 6:**
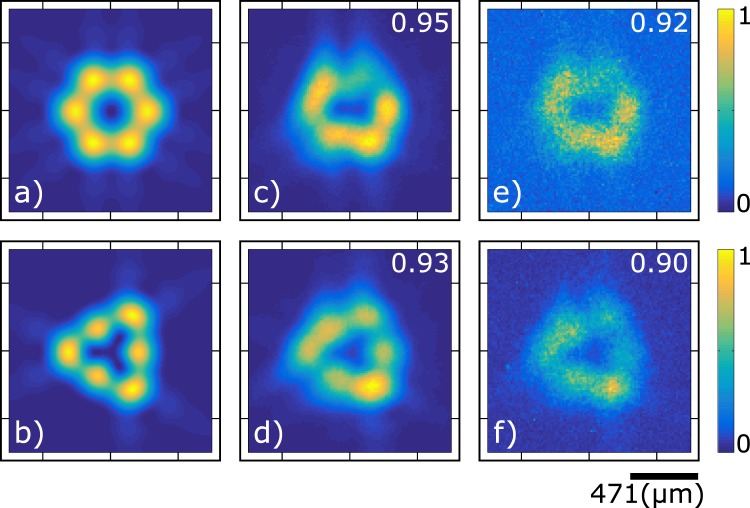
Intensity far-field diffraction patterns, at the single-photon level through a triangular aperture, of the signal photon. Numerical (first column), unconditioned data (second column) and conditioned data (third column).

In our experiment, see Fig. 2(e), we use a lens with an 8 mm focal length placed a short distance from the lantern to create an image of the lantern exit plane which fills a triangular aperture (TA; with 250 *μ* m side). The diffracted light is transmitted through a lens of 10 cm focal length placed at a distance of 10 cm from the aperture, itself placed 10 cm from the plane of our ICCD array. We record the single photon intensity pattern on this plane, both unconditioned and conditioned by the detection of the idler photon. Configuring both the incoherent and coherent experiments to produce a ‘doughnut’-shaped transverse intensity pattern, see Figs 4(h) and 5(h), we record the corresponding far-field diffractions patterns, both with our ICCD operating unconditioned and conditioned by the detection of the idler photon. The results are shown in Fig. 6, in which the first row corresponds to the incoherent addition experiment while the second row corresponds to the coherent addition experiment. In the first column we show simulations of the expected far-field diffraction patterns for both cases, in the second column we show the unconditioned transverse intensity measurements for the signal-mode single photons, while in the third column we show the corresponding conditioned measurements. Notwithstanding experimental imperfections, it is clear that there is an excellent agreement between simulations and measurements.

Let us note that while in the incoherent mode addition experiment, the far-field diffraction pattern exhibits a hexagonal pattern of lobes, in the coherent addition experiment the far-field diffraction becomes a triangular pattern with three lobes on each side. The latter result, signals the presence of OAM with topological charge 2 (or −2) in our heralded signal-mode single photons.

Relying on the coherent addition of modes, with arms of balanced lengths incoming into the MSPL, and by controlling the fraction of intensities in each of these two arms it becomes possible to obtain arbitrary coherent superpositions of the type cos*θ*|*u*_1_〉 + sin*θ*|*u*_2_〉 of two modes |*u*_1_〉 and |*u*_2_〉 associated to the two SMF inputs. This can serve as the basis for a half wave plate analogue (in modes |*u*_1_〉 and |*u*_2_〉 instead of two orthogonal polarization states) in the spatial degree of freedom with functionality at the single photon level^15,16^. The ability to controllably prepare single photons in a set of transverse modes, as we have demonstrated, is likewise of interest for the implementation of quantum key distribution protocols with larger alphabets (polarization implementations are limited to a dimension of 2)^31,32^.

**Figure 7:**
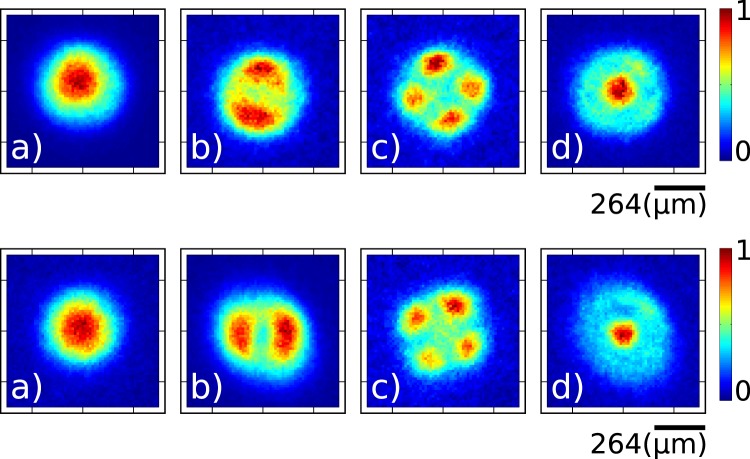
Transverse intensity at the single-photon level for the idler photon (636.6 nm; first row) and the signal photon (802.2 nm; second row) upon propagation over 400 m of few-mode fibre for one mode in each of the groups {*LP*_01_}, {*LP*_11*a*_, *LP*_11*b*_}, {*LP*_21*a*_, *LP*_21*b*_}, and {*LP*_02_}.

There are two further properties of our MSPL multiplexers operated at the single photon level, both likely to be of crucial importance in applications, that we wish to demonstrate: (i) the ability to transmit over hundreds of meters of fibre the prepared heralded single photons, and (ii) their inherent broadband nature. We have designed and performed an additional experiment in order to showcase these two capabilities. In particular, we have spliced a 400 m length of few-mode fibre to the MSPL output, with this MSPL placed to act on either the signal photon (802.2 nm) or the idler photon (636.6 nm). We have recorded with an ICCD camera the single-photon intensity distribution following transmission over 400 m. In Fig. 7 we show unconditioned experimental measurements for one output in each of the {*LP*_01_}, {*LP*_11*a*_, *LP*_11*b*_}, {*LP*_21*a*_, and *LP*_21*b*_} and {*LP*_02_} groups of modes which show mixing within each group due to cross-talk, upon propagation. In the first row we show counts for the idler photon and in the second row for the signal photon. These results make clear our ability to retain the prepared single-photon mode structure over this 400 m propagation distance, and also shows the operation at two spectral regions separated by >165 nm consistent with the inherent achromatic design of the MSPL.

## Conclusions

We have presented a demonstration of deterministic transverse mode conversion at the single photon level based on a spontaneous four wave mixing (SFWM) photon pair source and a mode-selective photonic lantern (MSPL). When using a single-mode MSPL input, we can convert the single-photon transverse profile from the fundamental mode to any of the six higher order modes *LP*_01_, *LP*_11*a*,*b*_, *LP*_21*a*,*b*_ and *LP*_02_. When using two MSPL inputs, we are able to obtain either the incoherent or coherent addition of two modes, amongst the six higher order modes guided in the system. Furthermore, the coherent nature of the addition, and extraction of usable orbital angular momentum, is demonstrated by far-field diffraction through a triangular aperture. Furthermore, we show that our MSPL is broadband, operating successfully at the 636.6 nm and 802.2 nm spectral locations of the idler and signal photons, respectively, and we show that our shaped single photons can be transmitted over a 400 m length of few-mode fibre. Our work has important implications for the future development of structured light experiments exploring quantum entanglement in the transverse momentum degree of freedom and for quantum key distribution protocols with a scalable alphabet dimension.

## Supplementary information

Control over the transverse structure of light at the single-photon level: Supplementary information

## Data Availability

The raw data used in our experimental plots is available upon request.
