# Supplementary material for: Control over the transverse structure and long-distance fiber propagation of light at the single-photon level
Source: Sci Rep. 2019 Jun 21;9:9015. doi: 10.1038/s41598-019-45082-6 (PMC6588692; doi:10.1038/s41598-019-45082-6)
Supplement: Supplementary file 1 — Control over the transverse structure of light at the single-photon level: Supplementary information [file 41598_2019_45082_MOESM1_ESM.docx]

Control over the transverse structure of light at the single-photon level: Supplementary information

D. Cruz-Delgado1, J.C. Alvarado-Zacarias2, H. Cruz-Ramirez1, J.E. Antonio-Lopez2, S. G. Leon-Saval3, R. Amezcua-Correa2, A. B. U’Ren1

1Instituto de Ciencias Nucleares, Universidad Nacional Auto ́noma de M ́exico, Apartado Postal 70-543, 04510 Cd.Mx., M ́exico 2CREOL, The College of Optics and Photonics, the University of Central Florida, Orlando, Florida 32816, USA 3Sydney Astrophotonic Instrumentation Laboratory,
School of Physics, University of Sydney, NSW 2006, Australia
(Dated: May 23, 2018)

**Mode Selective Photonic Lantern Fabrication**

In this work, we have fabricated mode selective photonic lanterns (MSPL) by inserting 6 dissimilar core diameter fibres into a fluorine-doped capillary with a refractive index Δn value of -9.54x10^-3^. After tapering, the input fibers and fluorine doped tube collapse and the resulting structure forms a few-mode waveguide, with the fused fibers acting as the new core and the low-index capillary serving as the cladding [1-3]. By utilizing dissimilar core sizes, it is possible to achieve mode selectivity [3-7], and thus map (on a one-to-one basis) an input fibre to one mode of the few mode output, Fig 1. In this case, the largest core size (largest propagation constant) evolves into the lowest order mode (LP01), while the smallest input core evolves into the highest order mode (LP02). The core diameters of the employed fibres are 1x23 μm, 2x18 μm, 2x15 μm and 1x11 μm addressing the LP01, LP11a,b, LP21a,b and LP02 modes respectively. The position of the fibres inside the fluorine-doped capillary is presented in Fig. 1(a). All input fibres have a graded index profile with maximum Δn = 16x10^-3^, and outer diameter of 125 μm. In the visible wavelength range, all six fibres support few modes but only the fundamental mode is excited in our experiments. Graded index fibres were chosen in order to reduce the taper length required to achieve an adiabatic transition [4, 7-9]. The fibres and fluorine-doped capillaries were fabricated in-house.

Once the fibres are inserted in the fluorine doped capillary, the bundle is adiabatically tapered using a CO2 laser tapering station AFL LazerMASTER LZM-100. In order to ensure an adiabatic transition, the tapering process is done in two stages. In the first stage, we apply a tapering factor of ~4.5, (from 1630 μm to ~350 μm outer diameter) with a linear transition of 1.5cm and a uniform waist of length equal to 5cm. During this process, the fibres are fused together creating a solid structure. In a subsequent step, the uniform (waist) section is inserted into a silica capillary with 370μm/820 μm inner/outer diameter respectively. This additional capillary is used to increase the outer diameter of the final multimode waveguide. By doing so, the resulting photonic lantern can be easily handled and can be directly spliced to a few mode transmission fibre. In a final tapering stage, the capillary is adiabatically tapered down to 125 μm final diameter. The resulting few mode fibre (FMF) core has a diameter of ~9μm, as shown in Fig. S1. The calculated V-number of at the FMF end are 7.06 and 8.89 for 802.2nm and 636.6 nm respectively. These values ensure that the MSPL supports 6 spatial modes at both wavelengths of interest. Finally, the MSPL is spliced to a 400 m long step index FMF with 12 μm core size, 86 μm cladding diameter and Δn ~6x10^-3^. The measured insertion losses are 1.5 to 2.5 dB insertion loss for 802.2nm and 636.6 nm respectively.


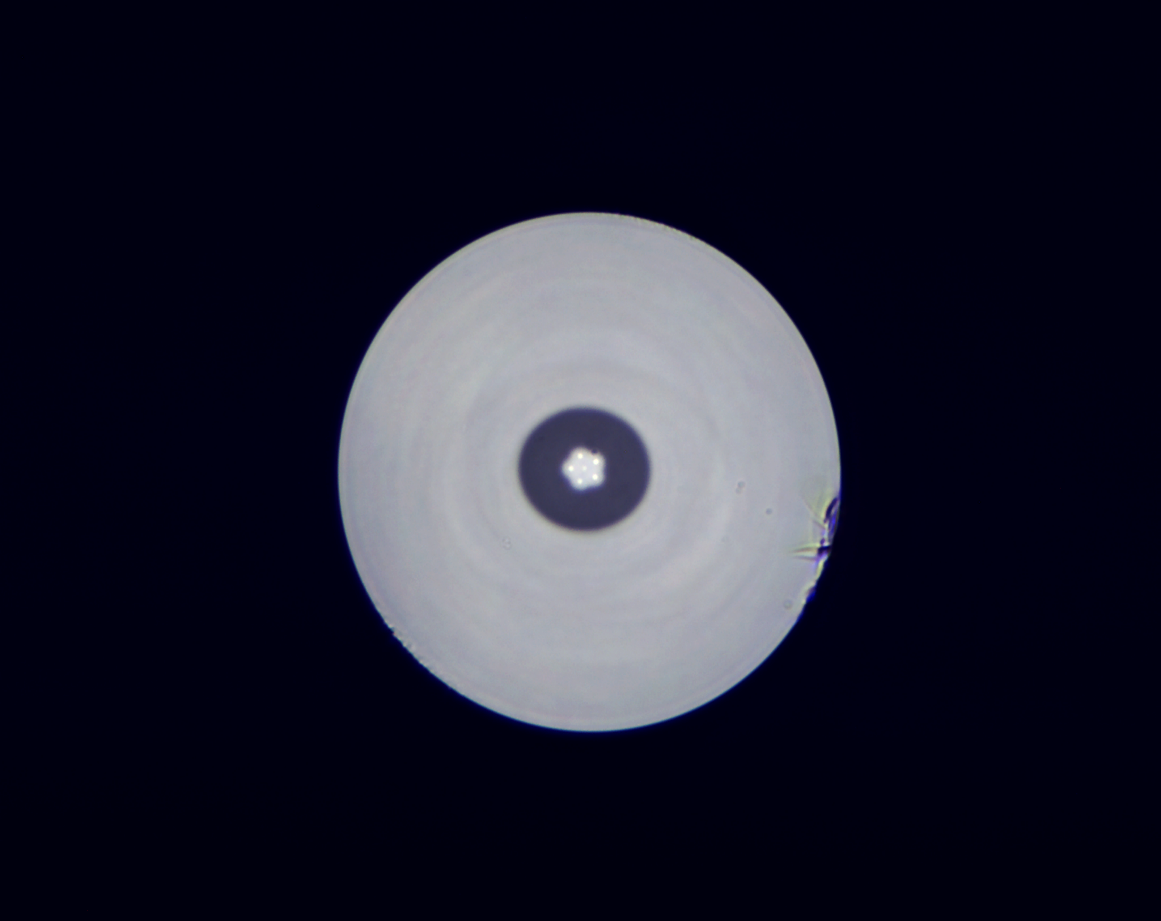


Fig. S1 Cross sectional view of the few mode fiber end of the fabricated MSPL, cladding diameter is 125 μm and core diameter around 9 μm. Dark region correspond to fluorine doped material.

1. T. A. Birks, I. Gris-Sánchez, S. Yerolatsitis, S. G. Leon-Saval, and R. R. Thomson, "The photonic lantern," Adv. Opt. Photon. 7, 107-167 (2015).
2. S. G. Leon-Saval, T. A. Birks, J. Bland-Hawthorn, and M. Englund, "Multimode fiber devices with single-mode performance," Opt. Lett. 30, 2545-2547 (2005).
3. S. G. Leon-Saval, A. Argyros, and J. Bland-Hawthorn, “Photonic Lanterns ”, Nanophotonics 2, 429 (2013).
4. Nicolas K. Fontaine, Roland Ryf, Joss Bland-Hawthorn, and Sergio G. Leon-Saval, "Geometric requirements for photonic lanterns in space division multiplexing," Opt. Express 20, 27123-27132 (2012).
5. S. Yerolatsitis, I. Gris-Sánchez, and T. A. Birks, "Adiabatically-tapered fiber mode multiplexers," Opt. Express 22, 608-617 (2014).
6. Sergio G. Leon-Saval, Nicolas K. Fontaine, Joel R. Salazar-Gil, Burcu Ercan, Roland Ryf, and Joss Bland-Hawthorn, "Mode-selective photonic lanterns for space-division multiplexing," Opt. Express 22, 1036-1044 (2014).
7. Leon-Saval, Sergio G., Nicolas K. Fontaine, and Rodrigo Amezcua-Correa. "Invited article: Photonic lantern as mode multiplexer for multimode optical communications." Optical Fibre Technology (2016).
8. Bin Huang, Nicolas K. Fontaine, Roland Ryf, Binbin Guan, Sergio G. Leon-Saval, R. Shubochkin, Y. Sun, R. Lingle, and Guifang Li, "All-fibre mode-group-selective photonic lantern using graded-index multimode fibers," Opt. Express 23, 224-234 (2015).
9. A. M. Velazquez-Benitez, J. C. Alvarado, G. Lopez-Galmiche, J. E. Antonio-Lopez, J. Hernández-Cordero, J. Sanchez-Mondragon, P. Sillard, C. M. Okonkwo, and R. Amezcua-Correa, "Six mode selective fiber optic spatial multiplexer," Opt. Lett. 40, 1663-1666 (2015).
